# A259 ASSESSING SYSTEM BASED QUALITY MEASURES IN THE CARE OF ACUTE PANCREATITIS AT A TERTIARY CARE HOSPITAL

**DOI:** 10.1093/jcag/gwab049.258

**Published:** 2022-02-21

**Authors:** A Saad Mohammed, F Almeshal, M Rai, L Hookey

**Affiliations:** 1 Queen’s University, Kingston, ON, Canada; 2 Gastroenterology, Queen’s University, Kingston, ON, Canada; 3 Gastrointestinal Diseases Research Unit, Queen’s University, Kingston, ON, Canada

## Abstract

**Background:**

Acute pancreatitis (AP) is an acute condition associated with significant morbidity and mortality. There has been a >75% increase in incidence since 1990 associated with annual Canadian healthcare costs greater than $200 million. Despite established guidelines for clinical management and quality indicators at the individual patient level, system-based performance measures are novel. Thus, hospital-based quality measures have recently been released by the American Gastroenterology Association (AGA) and are yet to be evaluated in a clinical setting.

**Aims:**

To assess rates of compliance to the AGA quality and performance measures in the care of acute pancreatitis.

**Methods:**

Retrospective chart review identified adult patients admitted with AP from June 2019 to May 2020 at Kingston Health Sciences Centre to review clinical profile. Data was collected, assessed, and stratified based on compliance with AGA performance measures of (1) cholecystectomy on index presentation of gallstone pancreatitis (GP), (2) initiation of early oral feeding within 24 hours, and (3) inappropriate parenteral nutrition (PN) use. Additional data collected included length of stay (LOS), presence of complications, and etiology of AP to assess for trends in associations. Descriptive and bivariate analysis was conducted.

**Results:**

There were 130 admissions of AP during the study period, of which the most common aetiologies were GP (52.3%) and alcohol (16.9%). Unknown/idiopathic AP had an incidence rate of 22.3%. The most likely etiology of having a prior presentation of AP was unknown/idiopathic AP (34.5%), closely followed by alcoholic AP (32%). The incidence of complications was 13.1% and median LOS was 4 days. 63% of eligible GP patients underwent cholecystectomy on index admission, whereas 20% proceeded with cholecystectomy at a later date. 88% of admissions had a diet ordered within 24 hours of admission. The median LOS was 4 days in the early feeding group, in contrast to 7.5 days in admissions which did not have diet ordered within 24 hours. A total of 3 patients received parenteral nutrition, however only one of these patients was eligible for enteral feeding.

**Conclusions:**

This study demonstrates that increasing cholecystectomy rates during index admission is an area to improve upon. Early diet orders were associated with a shorter hospital LOS; however additional evidence of direct correlation is required. Further studies are warranted to identify system level factors affecting quality of care as well as the development of targeted interventions in AP.

**Funding Agencies:**

None

